# Multiple Sclerosis and Viruses: « *Liaisons Dangereuses* »

**DOI:** 10.1002/rmv.70212

**Published:** 2026-10-04

**Authors:** Samanta Plavina, Daniela Malakovska, Jolanta Kalnina, Ilva Trapina, Elina Leonova, Mohammed Habib, Susanne Schifmann, Jegors Paramonovs, Nikolajs Sjakste, Yegor Vassetzky, Emmanuel Drouet, Natalia Paramonova

**Affiliations:** ^1^ Department of Pharmaceutical Sciences Faculty of Medicine and Life Sciences University of Latvia Riga Latvia; ^2^ Neurology Department Multiple Sclerosis Latvian Maritime Medicine Center Riga Latvia; ^3^ UMR 5075 CEA/CNRS/Université Grenoble Alpes Institut de Biologie Structurale Grenoble France; ^4^ Fraunhofer Institute for Translational Medicine and Pharmacology ITMP Frankfurt am Main Germany; ^5^ CNRS UMR9018 Université ParisSaclay Institut Gustave Roussy Villejuif France; ^6^ Koltzov Institute of Developmental Biology Moscow Russia

**Keywords:** autoimmunity, Epstein–Barr virus, multiple sclerosis, neuroinflammation, viral co‐factors, virus‐virus interaction

## Abstract

Multiple sclerosis (MS) is a complex, multifactorial neurodegenerative disease with an aetiology that is still partially elusive. Several risk factors are associated with MS development including genetic predisposition and various environmental stressors; however, recently, viral infections have gained significant attention as a crucial environmental trigger. Despite the well‐established link between Epstein‐Barr virus (EBV) and MS susceptibility, a single‐pathogen model fails to clearly characterise the molecular cascades driving the disease, suggesting that the transition from infection to autoimmune response involves more than one viral agent. In this review we examine the speculation that the interactions among different neurotropic viruses could serve a collective causative role in MS, which is under‐researched in the literature at present. We summarise contradictory evidence on the role of different viral agents, including the *Herpesviridae* family (Human Cytomegalovirus, EBV, Herpes Simplex Viruses HSV‐1 and HSV‐2, and Varicella Zoster Virus), *Paramyxoviridae* family (Measles Virus and Rubella Virus), Influenza virus, SARS‐CoV‐2, and Human Endogenous Retroviruses (HERVs). Through interplay within shared molecular pathways, immune modulation, and activation of latent infections, these viruses may collectively influence the inflammatory and demyelinating processes that characterise MS. This review evaluates the etiological role of viral interplay in MS through the cumulative and interactive effects of these pathogens. The available epidemiological, immunological, and experimental data converge on the concept that viral co‐exposures act synergistically to shape MS susceptibility in genetically predisposed individuals. Characterising these viral interactions presents a new research direction for the identification of novel biomarkers and the development of preventative strategies through the targeted control of viral networks.

## 1 Introduction

Multiple sclerosis (MS) is a chronic, immune‐mediated disorder of the central nervous system (CNS) characterised by demyelination, axonal damage, neuroinflammation, and progressive neurological decline [1, 2]. Demyelination causes a range of sensory and motor impairments, including mental disorders, cognitive deficits, fatigue, stiffness, intestinal and urinary system problems, and increased weakness [3].

Clinically, MS is characterised by recurrent episodes of neurological dysfunction, involving accumulation of chronic progressive neurological disability. This decline is commonly linked to accelerated brain atrophy, a process beginning as early as the initial stages of relapsing‐remitting MS (RRMS). Acute inflammatory relapses are linked to relapse‐associated worsening (RAW), while progression independent of relapse activity (PIRA) reflects a long‐term accumulation of disability unrelated to relapses, often attributed to chronic neurodegeneration [4]. Both RAW and PIRA contribute to disability accumulation, even during early stages of the disease [5]. PIRA becomes the dominant driver of disease worsening in progressive MS phenotypes: primary progressive MS (PPMS) exhibits background neurodegeneration from clinical onset, whereas secondary progressive MS (SPMS) follows the initial RRMS course due to continuous progression and depletion of neurological reserve [4, 6]. Diagnosis follows the 2024 McDonald Criteria, incorporating clinical and paraclinical evidence [7].

While the introduction of various disease‐modifying therapies (DMTs) has transformed MS management by reducing relapses and slowing disease progression, a cure is still lacking. Rather than stemming from an incomplete understanding of MS aetiology, this limitation reflects the challenge of developing DMTs targeting compartmentalised, smoldering neuroinflammation within the CNS, resulting in disease activity independent of relapse. Localised viral interactions are increasingly investigated as a key contributing factor. Current consensus is that MS results from a complex interplay between genetic predisposition and environmental triggers, among which viral infections, especially neurotropic viruses capable of crossing the blood‐brain barrier (BBB), acting as potential initiators or modulators of autoimmune responses in MS [8, 9, 10]. Indeed, viral nucleic acids, proteins or antiviral antibodies were detected in the blood, CSF or brain tissue [11, 12]. While nearly all MS patients are seropositive for Epstein‐Barr virus (EBV), a large‐scale longitudinal cohort study indicated that EBV infection precedes MS onset by years, with a 32‐fold increase in MS risk following late‐stage seroconversion in previously seronegative young adults [8]. Ability of EBV to establish lifelong latency in B cells, modulate host immunity, and engage in molecular mimicry with CNS antigens provides a mechanistic basis for its proposed involvement in MS pathogenesis [13]. In addition to EBV, other viruses, such Human Herpesvirus (HHV)‐6A, Cytomegalovirus (CMV), Herpes Simplex Virus (HSV)‐1/2, Severe Acute Respiratory Syndrome Coronavirus (SARS‐CoV)‐2, and endogenous retroviruses, might affect MS development or progression, either independently or through synergistic interactions with EBV. These viral agents can impact immune activation, oligodendrocyte survival, and BBB integrity, thereby facilitating demyelinating processes within the CNS [14, 15, 16, 17, 18]. In this review, we synthesise emerging data on the role of interaction between EBV and other viral agents in MS induction and progression. Understanding these interactions is critical for identifying novel diagnostic markers and therapeutic targets for MS.

## 2 Epidemiology and Mechanisms of MS

The global prevalence of MS is estimated at 1.9 million people, with an increasing incidence [19]. This rise in incidence is influenced by a combination of genetic, environmental, and geographic factors. Notably, MS is more common in North America and Europe, whereas lower prevalence rates are observed in regions such as Sub‐Saharan Africa and East Asia. The rising MS incidence likely reflects a combination of improved detection and environmental shifts acting on a susceptible genetic background. Advances in diagnostics, particularly Magnetic Resonance Imaging and updated McDonald criteria, have facilitated diagnosis, but do not fully account for epidemiological trends. Among environmental factors, the strongest evidence implicates EBV. Additional contributors include reduced early‐life microbial exposures (consistent with hygiene‐related immune dysregulation), lower Vitamin D levels linked to decreased sun exposure, and lifestyle factors such as smoking and adolescent obesity, all of which modulate immune function. Shifts in the gut microbiome may further influence systemic inflammation and autoimmunity. Their influence on systemic inflammation and autoimmunity is further compounded by ageing, which is associated with immunosenescence and a chronic low‐grade pro‐inflammatory state (‘inflammaging’) that may lower the threshold for autoimmune activation. The increasing female predominance also supports a role for sex‐specific environmental or hormonal influences. In contrast, there is no robust epidemiological evidence linking vaccination or recent epidemics to increased MS incidence.

The disease exhibits a strong sex disparity, affecting women significantly more than men. As of 2022, an estimated 74% of MS patients are female [20]. While MS is most frequently diagnosed between the ages of 20 and 40, there is a growing recognition of both paediatric and late‐onset MS cases [21]. Though MS itself is rarely fatal, severe disability associated with the disease can reduce life expectancy by approximately 7–14 years. Additionally, comorbidities such as cardiovascular disease and depression contribute to poorer outcomes [22].

Several risk factors are associated with MS development (Figure 1). They include a family history of MS, with specific human leucocyte antigen (HLA) genes strongly linked to MS susceptibility [23, 24]. Geographical trends suggest that MS is more prevalent in populations living farther from the equator, highlighting the potential role of vitamin D deficiency in disease development [25, 26]. Additionally, lifestyle factors such as smoking, obesity, diet and gut health and physical inactivity are associated with an increased risk of developing MS [27]. Recent data implicate viral infections, particularly EBV infection, to both the onset and progression of MS, reinforcing the hypothesis that MS may bet triggered by infections in genetically susceptible individuals [8, 13, 28, 29]. Indeed, EBV and other viruses such as HHV‐6 and Human Endogenous Retroviruses (HERVs) may contribute to the onset or progression of inflammation and neurodegeneration in MS via several biological mechanisms including mimicry, HERV activation, and viral transport to the central nervous system by immune cells. Moreover, this line of research has direct translational implications for developing preventive and therapeutic strategies, including EBV vaccination, antiviral therapies, and interventions targeting HERV activity.

**FIGURE 1:**
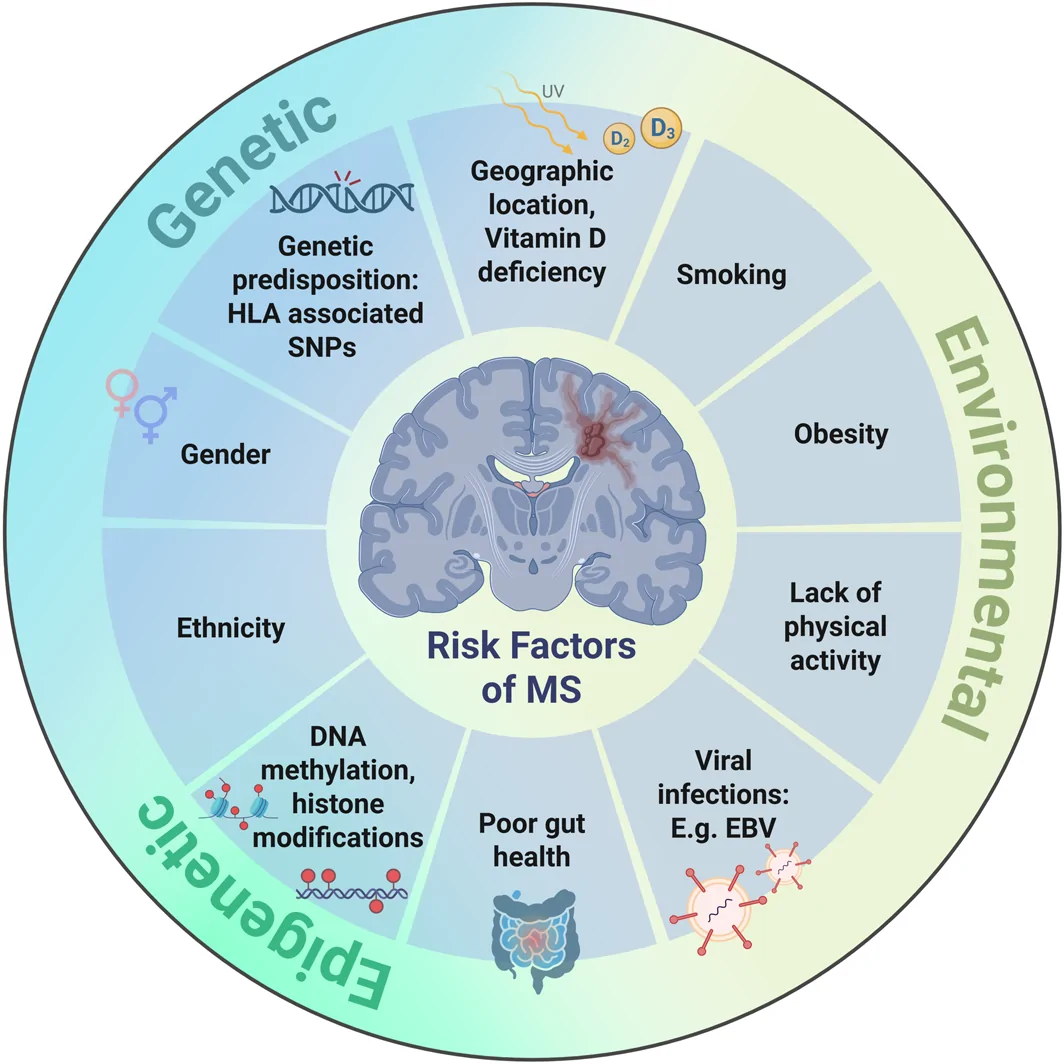
Key risk factors for developing MS. The increased susceptibility to MS involves a complex interplay between genetic susceptibility and diverse environmental triggers. Genetic predisposition is significantly conferred by SNPs within the HLA gene complex, alongside observed gender and ethnic differences in disease incidence. Key environmental and lifestyle risk factors include geographic location (often correlated with Vitamin D deficiency), smoking, stress, socioeconomic status, obesity and inadequate physical activity. Immunological triggers involve exposure to specific viral infections (e.g., EBV) and alterations in the gut microbiome. Ultimately, the integration of these risk factors is often mediated by epigenetic modifications including changes in DNA methylation and histone modifications which regulate gene expression and modulate the final risk profile. EBV, Epstein‐Barr virus; HLA, human leucocyte antigen; MS, multiple sclerosis; SNPs, single nucleotide polymorphisms. Created in https://BioRender.com.

Both DNA methylation and histone modifications mediate the interactions between genetic predisposition and environmental factors, contributing to the complex pathogenesis of MS [30]. Recent evidence from a case‐control study shows that EBV, HLA‐DRB1*1501 MS risk allele, lower vitamin D and low sun exposure share common differential DNA methylation profiles in pathways relevant to immune and neurological function [31].

## 3 Cellular and Molecular Mechanisms of MS Pathogenesis

### 3.1 Disruption of the Blood‐Brain Barrier

MS is triggered by activation of the CD4+ T‐helper cells Th1 and Th17 (Figure 2). Th1 and Th17 cells infiltrate the BBB and make it permeable for neutrophils. Th1 cells secrete pro‐inflammatory cytokines (IFN‐γ, TNF‐α), activating macrophages/microglia. Th17 cells produce IL‐17, IL‐21, and IL‐22, enhancing BBB permeability [32]. Oligoclonal bands qualitatively reflect the intrathecal IgG synthesis by B cells [33]. Overexpression and intracellular relocation of endothelial proteins vascular cell adhesion molecule‐1(VCAM‐1) and intracellular adhesion molecule‐1 (ICAM‐1) favours T‐cell migration into the brain [34]. At the same time, metalloproteinases secreted by immune cells digest proteins in the tight junctions disintegrating BBB [35]. Intrathecal IgG synthesis and IgG complement activation is observed, CNS IgG antibodies form IgG aggregates and bind complement C1q with high affinity, activating the classical complement pathway. This results in neuronal cell damage, which leads to neurodegeneration and demyelination [36, 37].

**FIGURE 2:**
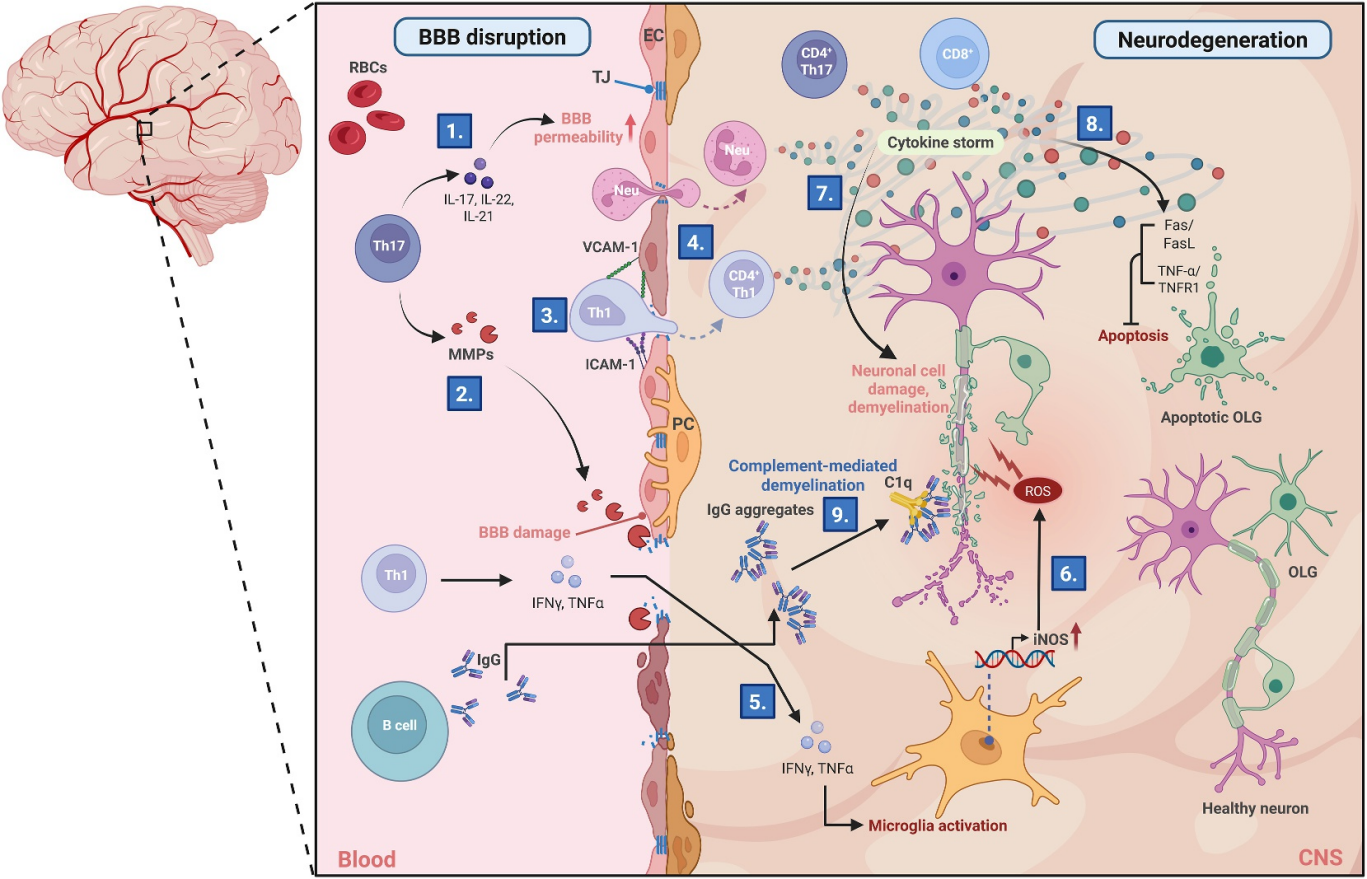
Key cellular and molecular mechanisms of MS pathogenesis within blood and central nervous system (CNS). BBB disruption: (1) CD4+T‐helper cell activation and pro‐inflammatory cytokine secretion responsible for BBB breakdown; (2) TJ protein degradation by MMP leading to BBB integrity loss; (3) Endothelial protein VCAM‐1 and ICAM‐1 overexpression facilitating T‐cell migration. Neurodegeneration and demyelination: (4) Immune cell migration through the BBB; (5) Activation of microglia; (6) Production of ROS promoting the destruction of myelin, OLG and neuronal cells; (7) Release of inflammatory mediators, ‘cytokine storm’ causing demyelination and neurodegeneration in the CNS; (8) OLG apoptosis through Fas/FasL or TNF‐α/TNFR1 signalling pathways; (9) Influx of IgG, including autoantibodies and complement mediated demyelination. BBB, Blood‐brain barrier; C1q, First subcomponent of the C1 complex of the classical pathway of complement activation; EC, endothelial cell; ICAM‐1, intercellular adhesion molecule 1; IFN‐γ, interferon‐gamma; IgG, immunoglobulin G; IL, interleukin; iNOS, inducible nitric oxide synthase; MMP, Matrix metalloproteinase; Neu, Neutrophil; OLG, Oligodendrocyte; PC, Pericyte; RBCs, Red blood cells; ROS, Reactive oxygen species; Th, T helper; TNF‐α, Tumour necrosis factor‐alpha; VCAM‐1, Vascular cell adhesion protein 1. Figure created with Biorender.com.

### 3.2 Neurodegeneration and Demyelination

The breakdown of the BBB is followed by the activation of immune cells, including T lymphocytes (CD4+ and CD8+), B lymphocytes, and neutrophils. CD4+ and CD8+ T lymphocytes directly attack oligodendrocytes and neurons, contributing to axonal damage. Meanwhile, B cells present antigens and secrete autoantibodies against myelin, leading to complement‐mediated demyelination. Within brain tissues, microglia expressing inducible nitric oxide synthase (iNOS) become activated, releasing nitric oxide and worsening oligodendrocyte lesions. In parallel, reactive oxygen species (ROS) are generated, further exacerbating cellular damage [38, 39, 40, 41].

The inflammatory response is amplified by a ‘cytokine storm’ [42]. Oligodendrocytes undergo apoptosis through Fas/FasL or TNF‐α/TNFR1 signalling pathways [43]. Additionally, mitochondrial dysfunction triggers excitotoxicity, characterised by excessive glutamate signalling. This leads to a cascade of damaging events, including calcium dysregulation, energy depletion, oxidative stress, and caspase activation [44].

Remyelination is impaired due to the inhibition of oligodendrocyte differentiation [45]. In progressive MS, sodium (Na^+^) homoeostasis is disrupted by the upregulation of voltage‐gated Na^+^ channel genes in white matter lesions. To counteract excessive neuronal excitability, a compensatory increase in voltage‐gated potassium (K^+^) channel gene expression occurs. Additionally, altered chloride (Cl^−^) homoeostasis‐marked by significant downregulation of voltage‐gated Cl^−^ channels in MS lesions‐may impair inhibitory neurotransmission and further contribute to neuronal hyperexcitability [46].

## 4 Viral Triggers of MS

EBV infection and immune reactions in a particular genetic context are currently considered to be decisive for the appearance of MS [8, 13, 28]; at the same time, there are abundant data on the association of other viruses with the pathogenesis and outcome of MS. Most of these viruses are known to have a tropism for the nervous system. As neurotropic pathogens can bypass the BBB and access CNS to directly induce myelin and nerve damage through viral cytotoxicity or immune dysregulation, it is hypothesised that viral exposure could drive neuroinflammation and progressive neuronal dysfunction, thereby promoting the onset of MS [47]. Furthermore, beyond direct injury, damage through immune system activation as a side effect of inflammation, might result in neurological symptoms [48]. Neurotropic viruses employ several mechanisms to invade the CNS (Table 1).

**TABLE 1:** Mechanisms of neuroinvasion by neurotropic viruses based on established primary or putative pathways for entry into the CNS.

Entry pathway in CNS	Description	Viruses	References
Axonal transport	CNS entrance mediated by axonal transport from infected peripheral neurons via the microtubule‐associated, retrograde transport mechanism	*HSV‐1* (Retrograde transport through trigeminal nerve from peripheral sites to the brainstem and other CNS regions) [49] *VZV* (Retrograde axonal transport from sensory ganglia to cerebral arteries [50])	[49, 50]
BBB or BCSFB disruption	Includes paracellular transport by altering tight junction proteins, transcytosis, direct infection of endothelial cells and immune‐mediated breach/weakening of BBB or BCSFB disruption	*SARS‐CoV‐2* (infection of BCSFB choroidal epithelial cells in choroid plexus as a potential entry site for SARS‐CoV‐2 into the CNS [51]) *EBV* (infection of brain endothelial cells modulates cytokines, chemokines and adhesion molecules inducing a local breach in the BBB [52]) *MV* (May cross the BBB by infecting brain endothelial cells [53])	[51, 52, 53]
‘Trojan horse’ via infected leucocytes	Transfer of pathogens to the CNS through infected peripheral immune cells	*SARS‐CoV‐2* (infected monocytes and macrophages possibly migrate through the BBB) [54] *EBV* (EBV‐infected B cell infiltration in the CNS) [55] *HIV‐1* (infected circulating monocytes and macrophages crossing the BBB) [56] *CMV* infected circulating leucocyte BBB infiltration in immunodeficient hosts (murine model) [57].	[54, 55, 56, 57]
Olfactory pathway	Retrograde transport via olfactory neurons to the CNS	*HSV‐1* (infection spreading from the olfactory epithelium to the olfactory bulb and subsequently to other brain regions, such as the limbic system) [58] *SARS‐CoV‐2* [59] *HHV‐6* [60] *Influenza* [61]	[58, 59, 60, 61]

Abbreviations: BBB, blood‐brain barrier; BCSFB, blood‐cerebrospinal fluid barrier; CMV, cytomegalovirus; CNS, central nervous system; EBV, Epstein‐Barr virus; HERV, Human Endogenous Retrovirus; HHV‐6, human herpesvirus 6; HIV‐1, human immunodeficiency virus 1; HSV‐1, herpes simplex virus 1; MV, measles virus; SARS‐CoV‐2, Severe Acute Respiratory Syndrome Coronavirus 2; VZV, Varicella‐Zoster Virus.

Below we will consider the contribution of specific viruses to MS pathogenesis.

### 4.1 Human Herpesvirus‐6 (HHV‐6)

HHV‐6 is a neurotropic virus that establishes lifelong latency and can reactivate under conditions of immunosuppression. It is primarily associated with roseola and, in some settings, encephalitis, whereas its potential involvement in multiple sclerosis (MS) remains incompletely understood. HHV‐6 comprises two closely related species, HHV‐6A and HHV‐6B [62]. Several studies have reported an association between HHV‐6A serological responses and MS, whereas a comparable association has not been consistently observed for HHV‐6B [63].

Numerous studies have investigated a possible association between HHV‐6 infection and MS. HHV‐6 seropositivity has been reported to be higher among patients with MS than healthy controls in some populations, including cohorts from Poland and Spain [64, 65]. Higher anti‐HHV‐6 IgG levels have also been associated with an increased risk of developing MS, as well as with clinical measures such as relapses and disability progression [66]. In addition, MS patients who are seropositive for HHV‐6 have been reported to exhibit higher circulating levels of inflammatory mediators, including TNF‐α, IFN‐γ, IL‐1β, IL‐6, and CCL‐5 [67]. These findings are consistent with an association between HHV‐6 infection or immune responses to the virus and inflammatory activity in MS; however, they do not establish whether HHV‐6 contributes directly to disease pathogenesis or whether increased viral reactivity reflects secondary immune activation associated with MS.

Studies specifically examining HHV‐6A have reported associations between high anti‐HHV‐6A antibody levels and MS risk that appear to be independent of the age at infection [68]. High HHV‐6A antibody levels have also been reported to interact with environmental and lifestyle factors, including smoking, low ultraviolet exposure, and vitamin D deficiency, in relation to MS risk [69]. Although these observations support a possible contribution of HHV‐6A to MS susceptibility, the observational nature of these associations does not establish a causal relationship.

Experimental studies provide evidence that HHV‐6 may directly affect neural cells. Both HHV‐6A and HHV‐6B have been shown in vitro to infect differentiated human neural cells, including glial cells and glutamatergic and dopaminergic neurons, with HHV‐6A producing more pronounced cytopathic effects than HHV‐6B [62]. These findings demonstrate that HHV‐6 can interact directly with neural cell populations; however, whether such infection occurs at a biologically relevant level in MS lesions and contributes to disease pathology remains unresolved.

The potential relationship between HHV‐6A and neuroaxonal injury has also been investigated. In a presymptomatic human cohort, elevated anti‐HHV‐6A antibody levels were associated with increased serum neurofilament light chain (sNfL), a biomarker of neuroaxonal injury [70]. This association raises the possibility that HHV‐6A‐related immune responses may be linked to early neuroaxonal damage, but it does not demonstrate that HHV‐6A is responsible for the observed injury.

Several experimental observations have suggested possible mechanisms through which HHV‐6A could influence neural function. Expression of the HHV‐6A latency‐associated transcript U94 A has been reported to impair cytoskeletal functions, including migration and maturation of oligodendrocyte precursor cells (OPCs), in vitro [71]. These findings provide a potential mechanism by which HHV‐6A‐related molecular activity could interfere with remyelination, although the relevance of this mechanism to MS in vivo remains to be established. Similarly, experimental expression of U94 A has been reported to affect neurite outgrowth and synaptic maturation, providing a further potential link between HHV‐6A and neuronal dysfunction [71]. Another HHV‐6A protein, U24, has been reported to interfere with the ubiquitin‐proteasomal system in neural cells and to interact with immune‐related proteins [72]. These experimental findings identify possible mechanisms of cellular dysfunction, but direct involvement of U24 in MS pathology has not been demonstrated.

Overall, current evidence supports an association between HHV‐6A immune responses and MS susceptibility and disease‐related biomarkers, while experimental studies demonstrate that HHV‐6A and its gene products can affect neural and immune‐related cellular functions. Nevertheless, the available evidence remains insufficient to establish HHV‐6A as a direct causal factor in MS. In particular, it remains unclear whether HHV‐6A infection contributes to disease initiation or progression, acts as a cofactor that modifies the effects of other infections such as EBV, or is preferentially reactivated as a consequence of MS‐associated immune dysregulation.

### 4.2 Epstein Barr Virus (HHV‐4)

The connection between EBV and MS has been a subject of extensive studies and reviews; here we will provide a short summary of recent published data. EBV is a double‐stranded herpesvirus ∼172 kilobases long that encodes over 85 proteins and 50 noncoding RNAs [73, 74, 75, 76]. It spreads mainly through saliva, first infecting cells in the mouth and then tonsillar lymphocytes [77, 78]. Once in the bloodstream, EBV establishes lifelong latency inside B cells [79].

The virus enters cells by attaching to specific receptors: in epithelial cells, it uses proteins like integrins and ephrin A2 [80]; in B cells, it binds to CD21 and interacts with MHC class II molecules [81, 82, 83]. Inside B cells, EBV can switch between different states of activity. In latency III, the virus expresses a set of latent genes (EBNAs and LMPs) that drive B cell growth and activation. In latency II, it expresses fewer genes, helping B cells survive while avoiding immune detection [84]. Latency I and 0 are even more restricted, with only a few noncoding RNAs or only one protein expressed (EBNA‐1), allowing the virus to hide in memory B cells [85, 86].

The latent cycle of EBV plays a crucial yet still debated role MS pathogenesis. Following primary infection, EBV establishes latency programs in B cells. During latency II/III, immunomodulatory proteins such as EBNA1, EBNA3A, LMP1, and LMP2A, are expressed in circulating B cells in both peripheral blood and CSF of MS patients, suggesting their involvement in immune dysregulation [87]. These latent proteins in turn might promote B‐cell survival and activation. As an example, it has been shown that LMP1 can function as a constitutively active receptor that mimics NF‐κB, JAK/STAT, and PI3K signalling pathways, enhancing proliferation, inflammation, and resistance to apoptosis. However, its detection in MS brain tissue remains highly conflicting across studies and is increasingly regarded as pathologically unconfirmed [88, 89]. Transcriptomic analyzes further support this role, showing increased expression of latency II/III and lytic‐associated viral genes in MS patients, alongside upregulation of inflammatory and antiviral host pathways, indicating that EBV influences the immune landscape [87]. Spatial imaging studies additionally demonstrate enrichment of EBV latency markers (e.g., EBNA1, LMP1) near immune cells within MS lesions, implicating EBV in localised neuroinflammation and potential neuronal stress [90]. Moreover, EBV persistence is facilitated by immune evasion mechanisms, as LMP2A‐mediated exploitation of PD‐1/PD‐L1, which may allow infected B cells to accumulate within the CNS and sustain chronic inflammation [91, 92].

As a leading mechanistic hypothesis, molecular mimicry may contribute via sequence homology between EBNA‐1 and several CNS proteins (e.g., GlialCAM, CRYAB, MBP, ANO2), promoting cross‐reactive immune responses [13, 93, 94, 95]. Additionally, EBNA‐1 might inhibit NK cell function, reduce apoptosis of infected B cells, and promote regulatory T‐cell migration, thereby sustaining a reservoir of infected and potentially autoreactive B cells [96, 97]. Other EBV nuclear antigens, particularly EBNA‐2, could further support B‐cell immortalisation and may enhance chronic antigen presentation, contributing to prolonged neuroinflammation in MS [98].

When infected B cells are activated, EBV can enter the lytic phase, producing new viral particles through a sequence of early and late gene activations. This reactivation is particularly relevant during primary infection later in life, when most EBV infections, which are typically asymptomatic in young children, instead manifest as infectious mononucleosis (IM), characterised by strong immune activation [99]. IM is marked by fever, swollen lymph nodes, fatigue, and high viral loads. During this phase, the immune system responds strongly, with natural killer (NK) cells and cytotoxic CD8+ T cells expanding to fight the virus. NK cells help control infected B cells, while CD8+ T cells recognise and destroy cells expressing viral proteins. After infection, EBV remains dormant in a small number of memory B cells, kept in check by immune surveillance.

Strong epidemiological evidence supports an association between EBV and MS. Nearly all people with MS show prior EBV infection, and IM significantly elevates long‐term risk [25, 100]. People with MS have higher levels of antibodies against EBV proteins, especially EBV‐encoded nuclear antigen‐1 (EBNA‐1), both in their blood and CSF [101, 102]. Epidemiological studies consistently demonstrate that patients with MS are almost universally infected with EBV and that the risk of developing the disease increases with the level of EBV‐specific antibody titres and these levels often rise before disease onset.

EBNA‐1 is essential for maintaining the viral episome in infected B cells, and EBNA‐1‐specific CD4+ T cells are key mediators of EBV immune control. In MS patients, EBNA‐1‐specific CD4+ T cells are significantly elevated compared with matched healthy EBV carriers, particularly in cohorts of untreated individuals stratified for MS‐associated HLA‐DR alleles. Using a panel of 51 overlapping peptides spanning the C‐terminal domain of EBNA‐1, these cells were shown not only to occur at higher frequencies but also to exhibit increased proliferative capacity and enhanced IFN‐γ production, reflecting a strongly polarised Th1 phenotype. Notably, this response appears highly specific to EBNA‐1, as T‐cell reactivity against other latent and lytic EBV antigens, as well as HCMV epitopes, does not differ between patients and controls, highlighting a distinct immunological signature in MS. Furthermore, MS patients exhibit a broader epitope recognition pattern across the EBNA‐1 C‐terminal domain, suggesting the presence of a highly sensitive and expanded autoreactive T‐cell repertoire capable of sustaining autoimmunity through molecular mimicry or bystander activation mechanisms [101].

Genetic factors also influence this relationship. The HLA‐DRB115:01 gene, the strongest genetic risk factor for MS, was associated with higher antibody levels to EBV and may make infection more efficient [103]. Some EBV genes, such as EBNA‐2, can interact with human immune genes and possibly alter immune regulation in ways that promote MS development [104, 105]. Studies of immune cells in MS show mixed results: some patients have more EBV‐specific CD8+ T cells, while others show signs of reduced function [106, 107]. Importantly, while EBV‐specific T cells and antibodies are often found in the CSF, direct evidence of active viral persistence within CNS brain lesions remains conflicting and inconsistently reproduced across studies [108].

In healthy individuals, EBV infection is kept under rigorous control by EBV‐specific immune responses, especially by cytotoxic CD8+ T cells, which eliminate proliferating and lytically infected B cells by targeting the various EBV‐encoded latent and lytic proteins. It is hypothesised that strong immune activation during IM, especially in genetically predisposed individuals, could lead to defective elimination of EBV‐infected B cells by cytotoxic CD8+ T cells (T cell exhaustion). Whether this acts as a direct causative factor or a secondary disease‐worsening mechanism remains debated. It may facilitate the migration of poorly controlled EBV + autoreactive B cells towards the CNS [109, 110]. Furthermore, an expansion of CD8+ T cells specific for EBV lytic antigens has been reported to occur during active disease in untreated MS patients, but not during periods of remission. This finding suggested a potential role for this lytic‐phase‐specific immune response in driving disease activity and progression [106].

Given the strong epidemiological and pathogenetic association between EBV and MS, there is increased interest in developing EBV vaccines as a preventive strategy. Prophylactic vaccine candidates aim to prevent EBV primary or late infection, which manifests as IM, thereby reducing the development of MS later in life. Several candidate vaccines are currently undergoing clinical evaluation for EBV and associated disease prevention, including a Phase 1 clinical trial (NCT06908096) [111] testing multivalent nanoparticle constructs (EBV gH/gL/gp42‐ferritin and EBV gp350‐ferritin) and a Phase 1/2 trial evaluating mRNA EBV vaccine candidate (NCT05164094) mRNA‐1189 [112]. However, given the widespread prevalence of EBV in the population, implementing such vaccines requires defined target populations—integrating the patient's genetic data (e.g., HLA risk alleles) and other environmental risk factors [113]. While current discussion focuses on prevention rather than treatment, the therapeutic potential of these vaccines is also being discussed. Notably, an ongoing Phase 2 clinical trial (NCT06735248) is currently evaluating an mRNA EBV vaccine candidate (mRNA‐1195) for relapse prevention in MS patients [114].

Recently published reviews discuss the efforts and potential for the introduction of EBV vaccines for MS prevention and therapy [113, 115]. However, candidate development is challenging due to complex aetiology of MS, difficulty of finding targets that would provide sufficiently strong cellular immunity, and the potential risk of unanticipated autoimmunity. Furthermore, decades‐long follow‐up, and high study‐related costs contribute to limited study development [113].

In conclusion, DNA viruses exhibit diverse interactions in MS aetiology, ranging from potential protective associations with CMV to compelling epidemiological evidence for EBV, although the precise extent and biological significance of EBV activity within the CNS remain a subject of active debate (Figure 3).

**FIGURE 3:**
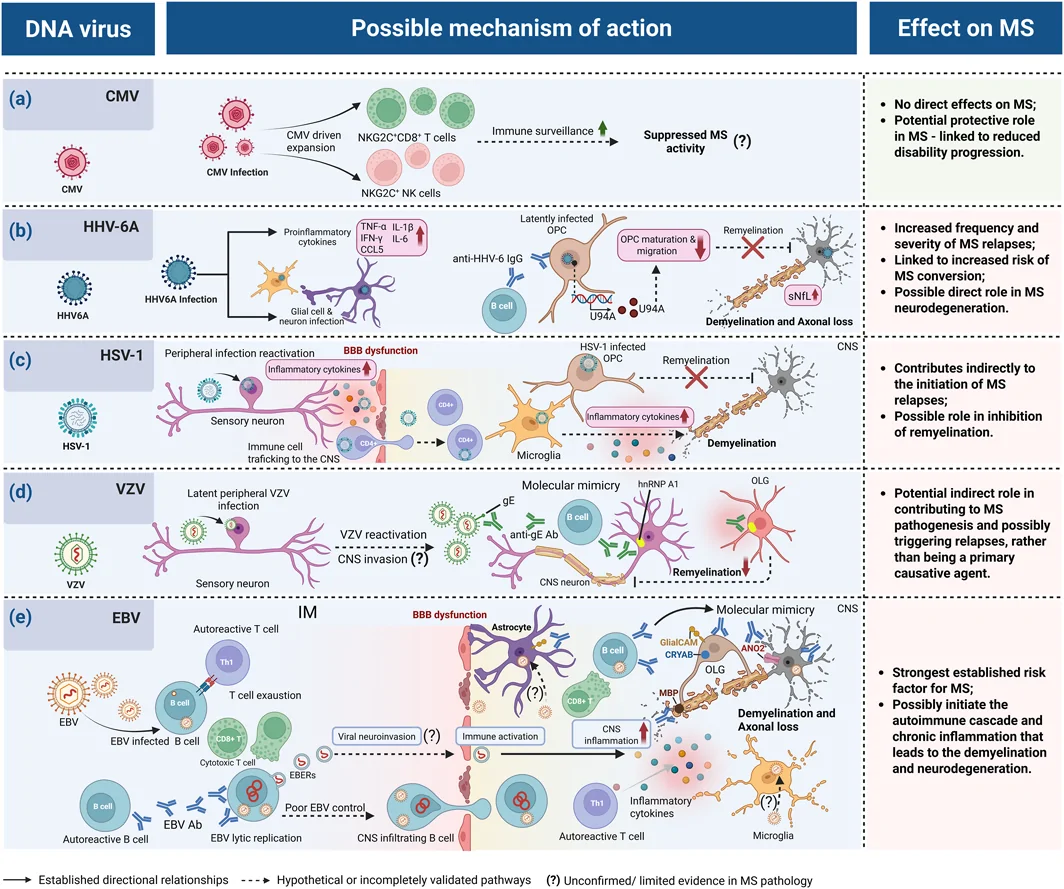
Neurotropic DNA viruses and their possible roles in MS pathogenesis and disease modulation. (a) CMV infection is associated with robust expansion of regulatory T‐cell populations and has been proposed to modulate immune responses in a manner that may reduce MS risk. (b) HHV‐6A seropositivity and elevated anti‐HHV‐6A antibody levels have been associated with MS risk and disease‐related measures, including neuroaxonal injury. Experimental studies indicate that HHV‐6A can infect glial and neuronal cells and induce cytopathic effects. The HHV‐6A U94 A transcript has been reported to inhibit OPC migration and maturation in vitro, providing a potential mechanism for impaired remyelination. Elevated anti‐HHV‐6A antibody levels have also been associated with increased sNfL levels, although whether this reflects a direct contribution of HHV‐6A to neurodegeneration remains unclear. (c) Peripheral HSV‐1 reactivation has been associated with systemic inflammatory responses and has been proposed to influence MS disease activity. (d) Latent VZV infection and its reactivation may influence immune responses relevant to MS; however, a direct causal role of VZV in MS pathogenesis has not been established. (e) Robust primary EBV infection (IM) and subsequent CD8+ T‐cell exhaustion enable the persistence of EBV‐infected autoreactive B cells. CNS neuroinvasion occurs via the direct trafficking of EBV‐infected B cells across the BBB or the translocation of EBV‐encoded RNAs (EBERs) via exosomes. Within the CNS, these factors drive neuroinflammation and B‐cell‐mediated antigen presentation, promoting the recruitment and survival of autoreactive T cells. This intrathecal immune activation triggers robust inflammatory cytokine production, exacerbating tissue injury. Furthermore, molecular mimicry between EBNA‐1 and host proteins (GlialCAM, MBP, CRYAB, ANO2) drives antibody‐mediated demyelination, while direct infection of neurons, glia, and endothelial cells contributes to localised neurodegeneration. Overall, the evidence for the involvement of these viruses in MS ranges from clinical associations to experimental observations and proposed mechanisms. Solid line: experimental evidence; dashed line: proposed/associative mechanism. ANO2, Anoctamin 2; BBB, Blood‐brain barrier; CCL‐5, C‐C motif chemokine ligand 5; CNS, Central nervous system; CRYAB, Alpha‐crystallin B chain; EBERs, EBV‐encoded small RNAs; EBNA‐1, Epstein‐Barr nuclear antigen 1; EBV, Epstein‐Barr virus; gE, Glycoprotein E; GlialCAM, Glial cell adhesion molecule; HHV‐6A, Human herpesvirus 6A; hnRNP A1, Heterogeneous nuclear ribonucleoprotein A1; HSV‐1, Herpes simplex virus type 1; IFN‐γ, Interferon gamma; IL‐1β, Interleukin 1 beta; IL‐6, Interleukin 6; IM, Infectious mononucleosis; MBP, Myelin basic protein; MS, Multiple sclerosis; NKG2C, Killer cell lectin‐like receptor subfamily C member 2; NK cells, Natural killer cells; OPCs, Oligodendrocyte precursor cells; sNfL, Serum neurofilament light chain; TNF‐α, Tumour necrosis factor alpha; U94 A, Human herpesvirus 6A protein U94; VZV, Varicella‐zoster virus. Figure created with Biorender.com.

### 4.3 Endogenous Retroviruses

Human Endogenous Retroviruses (HERVs) are ancient viral sequences integrated into the human genome that originate from past retroviral infections and may influence gene regulation and disease. They compose approximately 8% of the human genome [116] and most HERVs are silent due to epigenetic repression. An MS‐associated retrovirus (termed MSRV), a member of the HERV‐W family, has been particularly implicated in MS as MSRV particles and reverse transcriptase activity was detected in MS patients [117]. The HERV‐W envelope protein, also known as Syncytin‐1, has been implicated in amplifying the damaging effects observed in patients with MS; it can also serve as a biomarker for monitoring peripheral inflammation in MS [118]. Detected in the blood and brain, including brain‐infiltrating macrophages [119, 120], Syncytin‐1 is a powerful immune activator [121]. This activation likely amplifies the destructive potential of immune cells within the brain, leading to the destruction of oligodendrocytes and subsequent demyelination. Specifically, within MS lesions, this protein is found in microglia, where it is believed to be a driver of inflammatory damage to both myelin and neurons as demonstrated by the effects of pHERV‐W ENV within a myelinated co‐culture environment [122]. Additionally, HERV‐K gag and env proteins have been detected in lymphoid cells of MS patients [123].

Building on these findings, a humanised IgG4 monoclonal antibody targeting the MSRV envelope protein demonstrated potential neuroprotective effects in patients with relapsing‐remitting multiple sclerosis (RRMS).

However, MSRV neutralisation did not appear to have an effect on acute inflammation in MS [124]. This underscores that the role of these proteins in the pathogenesis of MS is not clearly established and their aberrant expression in MS patients may be a consequence of the disease. Indeed, HERV activation is associated with several neurological and psychiatric disorders [125] and HERV expression may be a consequence of MS‐associated inflammation rather than its cause [126]. Additionally, EBV infection may activate HERV expression [127]. The EBV‐HERV interaction in the context of MS will be discussed in more detail in the following sections.

Several models have proposed that activation of HERVs contributed to MS pathogenesis [128, 129]. Recent frameworks view MS as a non‐linear process shaped by interactions between environmental triggers and individual susceptibility factors. For instance, factors like vitamin D deficiency and EBV infection may exacerbate epigenetic dysregulation, further promoting HERV activation [130]. Environmentally induced HERV transactivation initiates expression of env and other viral proteins, which spread via exosomes and activate immune responses. Syncytin‐1 can stimulate toll like receptor 4 (TLR4), enhancing both innate and adaptive immunity, but it alone cannot explain the full disease complexity. Other HERV families, such as HERV‐K and HERV‐H, may be activated earlier, initiating cascades that recruit HERV‐W and amplify inflammation [131, 132].

Although HERV‐W proteins have been detected and are biologically active in MS, it remains unclear whether they are drivers or consequences of inflammation. Establishing definitive causality still remains a key limitation as most evidence is correlative or based on preclinical models. Compounding this issue, distinguishing MSRV from other HERV‐W elements is challenging due to their shared sequence homology. Furthermore, while HERW‐W proteins are strongly associated with disease biology, their activation in MS may simply reflect ongoing inflammation or EBV activity, making it difficult to isolate the specific contribution of MSRV alone. Depending on host genomic background, specific herpesvirus infections may trigger distinct HERV subsets, driving neuroinflammatory pathways and contributing to the heterogeneity of MS phenotypes.

Overall, RNA viruses exhibit varying degrees of interaction with the immune and nervous systems, influencing MS susceptibility and progression through distinct immunopathogenic pathways. While some exacerbate demyelination or trigger relapses, others like HIV appear to confer a paradoxical protective effect, underscoring the complex and multifactorial nature of virus–MS relationships (Figure 4).

**FIGURE 4:**
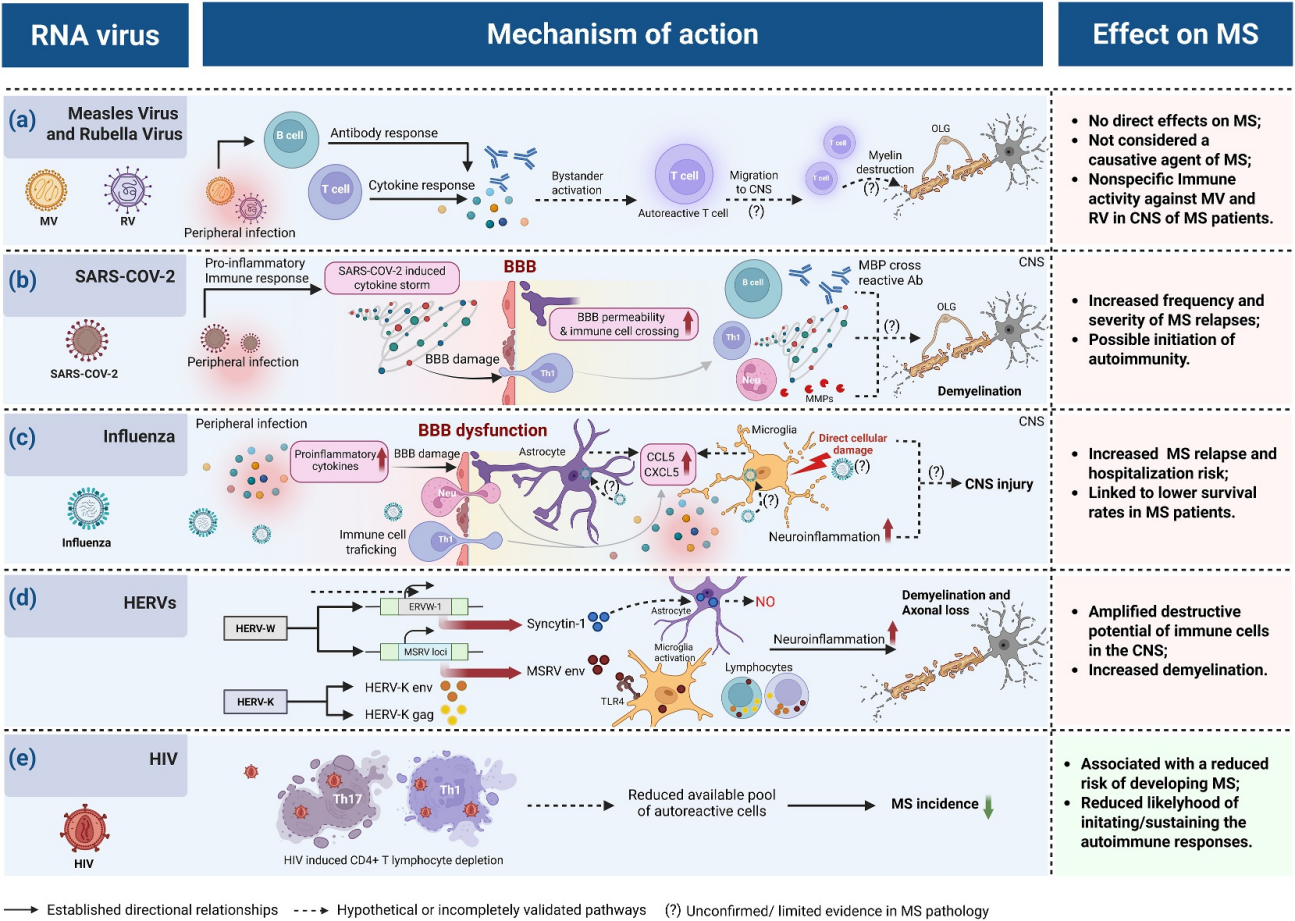
Neurotropic RNA viruses and HERVs and their possible roles in MS pathogenesis and disease modulation. (a) Peripheral measles virus and rubella virus infections can stimulate systemic immune responses that may promote bystander activation and trafficking of autoreactive T cells into the CNS, potentially contributing to myelin damage. (b) SARS‐CoV‐2 infection can induce systemic inflammatory responses, including increased circulating cytokines, which have been proposed to influence MS activity; the direct contribution of SARS‐CoV‐2 to MS pathogenesis remains uncertain. (c) Peripheral influenza infection can induce inflammatory cytokines and, in experimental models, cause cellular and tissue injury that may influence CNS inflammation. (d) Activation of HERV‐W and HERV‐K loci has been detected in association with MS. HERV‐W/Syncytin‐1 has been reported to activate inflammatory responses, including TLR4‐dependent signalling in experimental systems, and to affect microglial activation and OPC maturation. These findings provide potential mechanisms by which HERV‐W products could contribute to neuroinflammation and demyelination, although their causal role in MS remains uncertain. (e) CD4 depletion associated with HIV infection has been correlated with a reduced risk of developing MS, suggesting that alterations in immune surveillance may influence MS susceptibility. Solid line: experimental evidence; dashed line: proposed/associative mechanism. BBB, Blood‐brain barrier; CCL5, C‐C motif chemokine ligand 5; CNS, Central nervous system; CXCL5, C‐X‐C motif chemokine ligand 5; HERV, Human endogenous retrovirus; HERV‐K, Human endogenous retrovirus K; HERV‐W, Human endogenous retrovirus W; HIV, Human immunodeficiency virus; MBP, Myelin basic protein; MMP, Matrix metalloproteinase; MS, Multiple sclerosis; SARS‐CoV‐2, Severe acute respiratory syndrome coronavirus 2; Th1, Type 1 helper T cell; TLR4, Toll‐like receptor 4. Figure created with Biorender.com.

### 4.4 Human Cytomegalovirus (CMV)

CMV is a Herpesvirus (HHV‐5) that establishes lifelong latency, often remaining asymptomatic in healthy individuals but posing serious risks to immunocompromised patients and newborns. Recent studies suggest a potential protective role of CMV infection in MS. Indeed, high levels of HCMV antibodies in MS patients are associated with lower levels of serum neurofilament light chain (sNfL), a biomarker of neuroaxonal damage and disability progression [133]. Additionally, MS patients with elevated HCMV antibody levels tend to require first‐line treatment at a later stage, suggesting a slower disease course [134]. In CMV‐infected MS patients, natural killer (NK) cells, CD8+ T cells, and NKT‐like cells exhibit altered phenotypes and increased expression of the NKG2C receptor. These immune modifications appear to contribute to reduced disability progression in MS [135].

### 4.5 Other Viruses

#### 4.5.1 Herpes Simplex Virus (HSV‐1 and HSV‐2)

HSV‐1 and HSV‐2 are members of the *Herpesvirida*e family. These viruses have the capacity to establish lifelong latency within the nervous system, periodically reactivate, and directly interact with neural cells [136].

Oligoclonal IgG bands directed against HSV‐1 in the CSF of patients with MS have been reported [137]. While anti‐herpesvirus therapy was associated with a reduced number of new active MRI‐detected lesions in a small MS cohort study [138], a mechanistic role for HSV‐1 in impaired remyelination remains speculative, relying solely on in vitro models demonstrating direct HSV‐1 infection of human oligodendrocyte progenitor cells [139].

Meta‐analyses of case‐control studies on the seroprevalence of IgG antibodies against HSV‐1 and HSV‐2 in MS patients have yielded mixed results. While some demonstrated a significant association between viral antibodies and MS, others showed a lack of association [138, 139], suggesting a secondary role for these viruses in MS pathogenesis, mediated through indirect immune mechanisms or by interaction with other viruses [140] and activation of HERV‐W in cells involved in MS pathogenesis [141, 142]. Similarly, attempts to detect viral DNA in MS patients CSF and PBMCs versus controls have yielded low prevalence rates (< 5%) and no significant differences when compared to patients with other neurological disorders [143]. Moreover, a recent mendelian randomisation study did not reveal any effect of HSV on the risk of MS [18].

Preclinical animal models suggested that prior exposure to HSV‐1 may contribute to the development and symptom severity of MS [144], leading to the hypothesis that peripheral HSV‐1 reactivation during acute attacks might act as a nonspecific trigger for MS relapses rather than a primary aetiology [145]. Proposed indirect mechanisms of HSV action include bystander immune activation potentially resulting in proinflammatory cytokine production by CD8+ T cells (IL‐6, IFN‐γ, TNF‐α) and microglia (IL‐6, IL‐8, CCL5 and CXCL10), with increased CD4+T cell recruitment to the brain [144, 146, 147]. However, the contribution of these mechanisms to MS pathology remains uncertain.

One of the proposed demyelination mechanisms was molecular mimicry; a mimicry between an epitope shared by the HSV‐1 glycoprotein gB and a MS17 cross‐reacting brain factor was reported [148]. In addition, a TCR that recognized both HSV‐1‐encoded peptide and MBP was isolated from an MS patient [149]. Although HSV‐1 can invade the CNS and cause acute encephalitis with direct tissue injury [16, 150, 151], whether it contributes to direct CNS damage in multiple sclerosis remains hypothetical.

#### 4.5.2 Measles Virus and Rubella Virus

Measles virus (MV) and rubella virus (RV) are highly contagious viruses that belong to different viral families but share a common mode of transmission through respiratory droplets. MV, a member of the *Paramyxoviridae* family, causes a systemic infection characterised by fever, rash, and transient, generally reversible immunosuppression associated with immune amnesia, whereas RV, a member of the *Togaviridae* family, typically causes a mild illness but poses a serious risk to foetal development when infection occurs during pregnancy [152].

Controversial results on the presence of measles virus RNA in the brains of MS patients exist [153]. Moreover, population‐based studies found no significant associations between MS and previous measles infection [150, 154]. On the contrary, a recent study demonstrated that seropositivity towards RV was associated with an increased risk of MS for unvaccinated subjects [151]. These findings are inconsistent across methodologies (molecular detection vs. epidemiology), suggesting possible cohort bias, or indirect associations rather than a direct causal role.

One possible mechanism for involvement of these viruses is a molecular mimicry of viral proteins to human ones. MV structural proteins share some similarity to the myelin basic protein (MBP), although MBP‐specific T‐cells exhibit no cross‐reactivity with MV [152]. Thus, structural similarity alone is insufficient for autoimmune activation.

#### 4.5.3 SARS‐COV‐2

SARS‐CoV‐2 is a single‐stranded RNA virus belonging to the *Coronaviridae* family, responsible for the COVID‐19 pandemics that began in late 2019 [155]. COVID‐19 is not a direct cause of MS. To date, there is no solid evidence that SARS‐CoV‐2 can trigger MS in healthy persons [156].

While the respiratory tract is the primary route of entry for SARS‐CoV‐2, some evidence shows that the virus can invade the CNS leading to neurological symptoms and cognitive dysfunction [157, 158]. This observation prompts consideration of the virus's potential role in modulating disease progression in MS patients. Several studies have addressed this hypothesis speculating that SARS‐CoV‐2 may be involved in triggering MS relapses in previously affected individuals thus exacerbating demyelination processes as a result of pro‐inflammatory reaction [159, 160]. The proposed indirect mechanisms remain largely theoretical and include systemic and CNS inflammation driven by a SARS‐CoV‐2‐induced cytokine storm, molecular mimicry resulting in the potential cross‐reactivity of SARS‐CoV‐2‐specific T cells with myelin antigens, and the upregulation of matrix metalloproteinases (MMPs) [161, 162, 163, 164]. SARS‐CoV‐2‐induced CNS demyelinating lesions have also been reported in clinical case reports involving non‐MS patients [165, 166]. In vitro models of the human BBB suggest that SARS‐CoV‐2 activity in the immune periphery and CNS microenvironment may potentially reduce BBB integrity and facilitate the infiltration of peripheral immune cells into the CNS. While early in vitro studies suggested that SARS‐CoV‐2 might influence long‐term MS risk [167, 168], current evidence does not support SARS‐CoV‐2 as an initiating factor in MS development.

While SARS‐CoV‐2 RNA has been detected in CNS in some cases, this appears to be relatively uncommon and is rarely associated with evidence of viral neuroinvasion [167, 169]. This suggests that indirect mechanisms most likely drive these neurological complications.

Viral infections (including COVID, influenza, shingles) may increase the risk of pseudo‐flare‐ups [170]. One study suggested an increased MS disease activity after severe COVID [171]. Although cases of MS reactivation have been reported, post‐vaccination MS reactivation or flare‐ups are extremely rare [172].

There is no convincing evidence that SARS‐CoV‐2 initiates MS. However, systemic infection may precipitate pseudo‐flares or, less commonly, serve as a non‐specific trigger for disease exacerbation.

Indeed, distinguishing true disease causation versus transient immune activation is difficult, especially in observational and post‐infection studies.

#### 4.5.4 Influenza Virus

Influenza, commonly known as the ‘flu’, is a highly contagious respiratory infection caused by Influenza Viruses belonging to the family of *Orthomyxoviridae*; it can affect people of all ages. Symptoms include fever, feeling chills, headache, weakness, sore throat; however in high‐risk individuals, infection can result in life‐threatening complications [168, 173]. In light of findings in the Lewis rat model of EAE that implicate the lungs as a repository site for myelin‐reactive effector cells [174], along with epidemiological evidence linking influenza infection to increased MS relapse and hospitalisation risk [175, 176], a possible connection between respiratory viral infections and MS pathophysiology has been suggested.

Indeed, clinical surveys show that influenza infection increases relapse risk in patients with RRMS [177]. Furthermore, a retrospective cohort study identified pneumonia and influenza as significant predictors of reduced survival among patients with MS [178]. Although it remains unclear whether increased mortality reflects direct influenza‐associated respiratory complications or indirect effects mediated by infection‐induced disease exacerbation, a potential mechanistic link is supported by findings from a murine EAE model demonstrating impaired pulmonary immunity and subsequent uncontrolled viral replication [179]. Despite the implications of comorbidity, surprisingly little is known about the specific paths of interaction between MS and Influenza Virus. It may potentially involve up‐regulation of inflammatory cytokines both peripherally and centrally within the CNS. Peripheral influenza infection causes upregulation of immune response genes downstream of type I and type II IFN receptor signalling and in CNS, together with and upregulation of chemokine CXCL5 [180] which could further enhance inflammatory BBB dysfunction elevating MS attacks [181].

In murine EAE models, prior influenza virus infection may promote type 1 T cell recruitment to the CNS through CCL5 upregulation, providing a potential mechanistic framework for how systemic immune responses may indirectly contribute to neuroinflammation [182].

Furthermore, experimental studies show that influenza exposure can activate glia cells and potentially epithelial cells lining BCSFB, thus potentially facilitating peripheral immune cell trafficking to the CNS during clinical exacerbations [180]. Beyond these indirect mechanisms, in vitro models suggest that viral infection of microglia and astrocytes may induce direct cellular injury, proinflammatory cytokine production, and apoptosis [183]. However, whether direct glial infection occurs in MS or contributes to disease‐associated neuroinflammation in MS remains hypothetical.

#### 4.5.5 Human Immunodeficiency Virus (HIV)

HIV is a retrovirus that primarily infects cells of the immune system, notably CD4+ T lymphocytes, leading to progressive immune suppression. By integrating into the host genome, HIV establishes a chronic infection that, if untreated, can lead to Acquired Immunodeficiency Syndrome (AIDS). The virus is transmitted through blood, sexual contact, and from mother to child during childbirth or breastfeeding. Beyond its well‐known role in immune deficiency, HIV has complex interactions with various physiological systems, including the central nervous system, and its presence can influence the course of other immune‐mediated conditions. Some evidence suggests that HIV infection may be associated with a reduced risk of developing MS [184, 185]. HIV targets CD4+ T cells also implicated in the autoimmune attack on myelin in MS. By depleting these cells, HIV may inadvertently lower the likelihood of initiating or sustaining the autoreactive immune responses that characterise MS. However, these cells play crucial roles in overall immune function, so their loss can lead to significant immunodeficiency and susceptibility to opportunistic infections. On the other hand, people treated with combined antiretroviral therapy (cART) that reconstitutes normal CD4+ T cell levels also have a reduced risk of MS [186].

#### 4.5.6 Varicella Zoster Virus

Varicella Zoster Virus (VZV), another neurotropic Herpesvirus (HHV‐3), causes chickenpox upon primary infection and can reactivate later in life as shingles (herpes zoster), particularly in immunocompromised individuals or the elderly. The MRZ reaction (MRZR), composed of the three antibody indices against measles, rubella and VZV, has been found positive in the majority of RRMS patients: in different populations, 50%–70% of MS patients were positive for MRZ antibodies [187]. Intrathecal MRZ reaction is frequently associated with inflammation [188]. Clinical genetic studies suggest the development of the MRZ reaction is partially determined by host's genetic profile and associated to a specific HLA allele (HLA‐DRB1*15:01) [181].

VZV establishes latency in the nervous system and reactivates under immunosuppressive conditions and psychological stress [182]. A case study documented the transition from VZV‐caused encephalitis to autoimmune encephalitis and subsequently to MS [189]. Interestingly, VZV reactivation may occur in some MS patients receiving dimethyl fumarate, anti‐CD20 therapies and cladribine, potentially reflecting the immunosuppressive effects of these treatments, particularly lymphocyte depletion [190, 191, 192, 193, 194].

Neurological complications of VZV reactivation, including encephalitis, myelitis, meningitis, and neuropathy, may mimic or exacerbate MS symptoms [195]. However, although several studies suggest an association between VZV reactivation and MS relapses, a causal relationship remains uncertain [196, 197].

VZV glycoprotein E (gE) shares > 62% amino acids sequence similarity with a Prion‐like domain (PrLD) of HNRNPA1, a human protein involved in pathogenesis of the MS [198]. In vitro studies demonstrate that HNRNPA1 autoantibodies from MS patients can enter neurons and induce stress granules [199]. Moreover, mutations in HNRNPA1, which cause its mislocalisation and presentation on the surface of neuronal cells, were associated with an increased susceptibility to neurodegeneration [200].

## 5 Interaction of EBV and Other Viruses in the Context of MS

A closer look into the synergistic or antagonistic interactions between EBV and other neurotrophic viruses implicated in MS may provide a mechanistic basis for the epidemiological synergy reported in the studies. The cumulative pathogenic effect of multiple viral exposures, potentially mediated by direct molecular cross‐talk, may hold greater significance than the contribution of a single virus (Figure 5).

**FIGURE 5:**
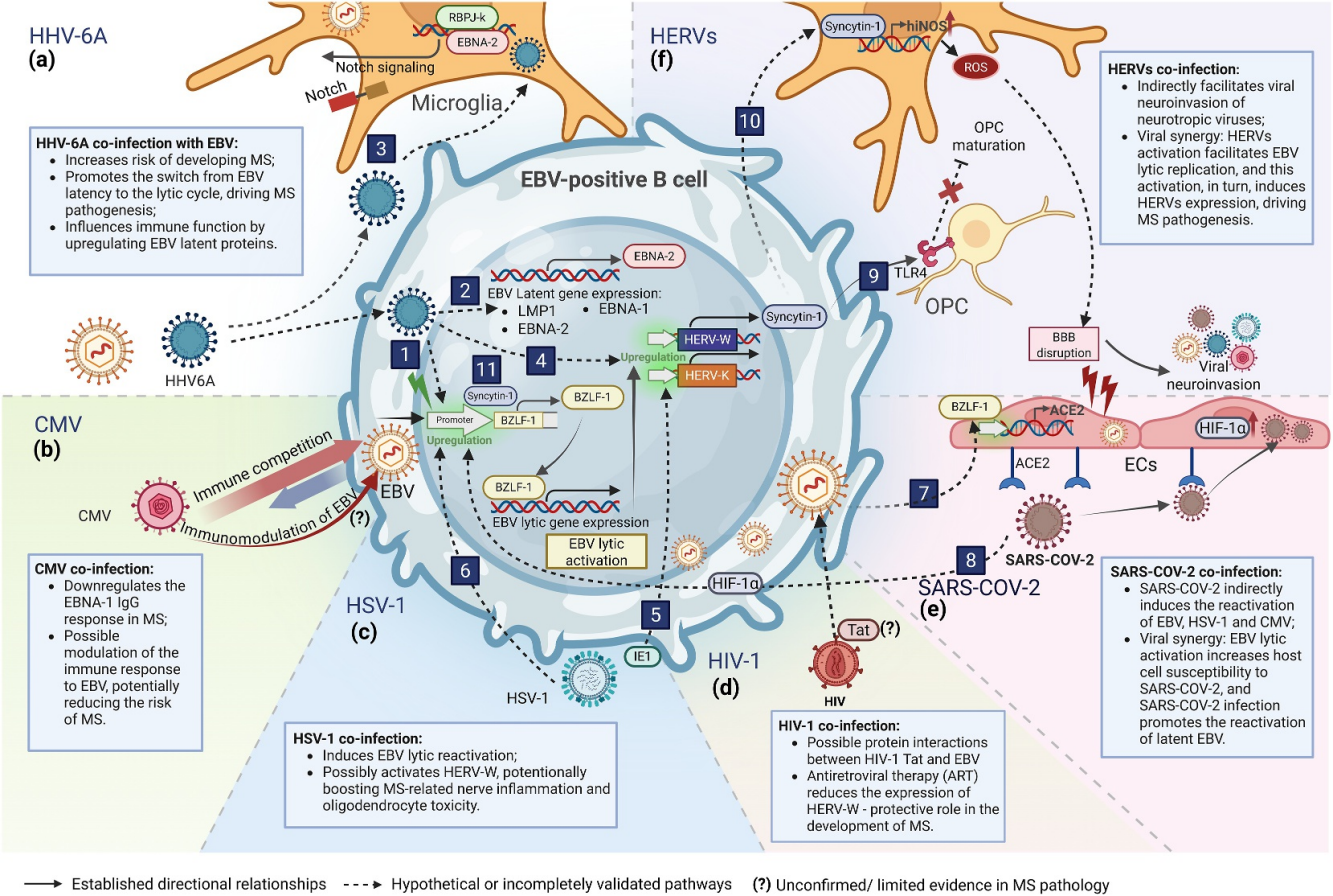
Proposed viral co‐interactions involving EBV, HHV‐6A, CMV, HSV‐1, HIV‐1, SARS‐CoV‐2 and HERVs in relation to MS. (a) Experimental studies suggest that HHV‐6A can interact with EBV‐positive B cells and may influence EBV lytic and latent gene expression. 1. HHV‐6 A infection of EBV‐positive B cells has been reported to increase BZLF1 promoter activity, potentially facilitating EBV lytic gene expression. 2. HHV‐6 A has also been reported to enhance expression of the EBV latent proteins LMP1 and EBNA‐2 in experimental systems. 3. EBNA‐2 interacts with cellular RBPJ‐κ, a component of the Notch signalling pathway, and may thereby influence cellular functions relevant to immune and glial responses. 4. HHV‐6 A infection has been reported to increase HERV transcription in experimental systems. These interactions represent proposed mechanisms and their relevance to MS pathogenesis remains to be established. (b) CMV infection has been proposed to modulate antiviral and immune responses that could influence EBV‐associated effects and MS risk; the direction and clinical relevance of this interaction remain uncertain. (c) Experimental studies indicate that HSV‐1 can influence HERV‐W transcription and EBV lytic reactivation, suggesting possible interactions between herpesviruses and endogenous retroviral elements. (d) Experimental and clinical observations have suggested possible interactions between HIV‐1 Tat and EBV that could contribute to the altered MS risk observed in people living with HIV; however, the mechanisms underlying this association remain incompletely defined. (e) SARS‐CoV‐2 infection has been associated with EBV reactivation in some experimental and clinical settings, raising the possibility that such interactions could influence inflammatory responses relevant to MS. (f) HERV‐W/Syncytin‐1 has been proposed to contribute to inflammatory processes relevant to MS. 9. Experimental studies have reported that Syncytin‐1 can activate TLR4‐dependent inflammatory signalling and impair OPC maturation, providing a potential mechanism that may contribute to demyelination. 10. Syncytin‐1 has also been reported to increase iNOS expression in microglia in experimental systems. Whether these effects constitute a causal contribution of HERV‐W/Syncytin‐1 to MS pathogenesis remains unresolved. Solid line: experimental evidence; dashed line: proposed/associative mechanism. ACE2, Angiotensin‐converting enzyme 2; BBB, Blood‐brain barrier; BZLF1, Epstein‐Barr virus early antigen R (immediate‐early protein); CMV, Cytomegalovirus; CNS, Central nervous system; EBNA‐2, Epstein‐Barr nuclear antigen 2; EBV, Epstein‐Barr virus; HERVs, Human endogenous retroviruses; HERV‐W, Human endogenous retrovirus type W; HHV‐6A, Human herpesvirus 6A; HIF‐1α, Hypoxia‐inducible factor 1‐alpha; hiNOS, Human inducible nitric oxide synthase; HIV‐1, Human immunodeficiency virus type 1; HSV‐1, Herpes simplex virus type 1; IE1, Immediate‐early protein 1; LMP1, Latent membrane protein 1; LTR, Long terminal repeat; MS, Multiple sclerosis; OPC, Oligodendrocyte precursor cell; RBPJ‐κ, Recombination signal binding protein for immunoglobulin kappa J region; SARS‐CoV‐2, Severe acute respiratory syndrome coronavirus 2; Tat, *Trans*‐activator of transcription; TLR4, Toll‐like receptor 4. Figure created with Biorender.com.

### 5.1 EBV and HHV‐6A

HHV‐6A interacts with EBV by infecting EBV‐positive B cells, upregulating the BZLF‐1 promoter, and therefore initiating the EBV lytic cycle [201, 202]; this may trigger an adverse immune reaction causing MS. A recent nested case‐control study, that included biobank samples collected before the clinical onset of MS, revealed that seropositivity for HHV‐6A was significantly associated with the level of sNfL. Furthermore, sNfL levels were significantly higher in cases with combined seropositivity for HHV‐6A and EBV thus indicating that the interplay between EBV and HHV‐6A was a key aspect of their combined contribution to MS risk [17]. While late‐onset EBV infection resulting in IM may be sufficient to trigger MS without the need for additional viral stimuli, early‐onset EBV infection might require a second hit by HHV‐6A infection to activate EBV lytic cycle that triggers MS [17, 68]. Consequently, co‐infection of HHV‐6A and EBV could result in a 6.7 fold increased risk of developing MS [17]. In addition, HHV‐6A upregulated the expression of EBV growth‐transformation‐associated latent proteins LMP1 and EBNA‐2 [203]. HHV‐6 A induced EBNA‐2 directly interacts with the cellular DNA‐binding protein of the Notch signalling pathway RBPJ‐κ, affecting function of both immune and glial cells [204].

### 5.2 EBV and CMV

Positive correlations exist in relation to MS risk between CMV serostatus and the EBV EBNA‐1 antigen, as well as between CMV and HHV‐6A. Specifically, individuals who were CMV seronegative but seropositive for either EBNA‐1 antigen (amino acid 385–420) or HHV‐6A had the highest risk of developing MS, highlighting a potential protective role of CMV against the disease [205]. This effect may be attributed to immune competition between CMV and EBV, or to CMV‐mediated modulation of immune control over EBV [206, 207].

The hypothesis that certain viral co‐infections might mitigate MS risk remains a significant, yet poorly understood, gap in current literature. While clinical data increasingly associate CMV and HIV seropositivity with a reduced susceptibility to MS, evidence for protective effects of other viruses remains limited [186, 205, 208].

The role of CMV in MS risk reduction may be related to immune competition with EBV and an overall enhancement of immune system reactivity against EBV and possibly also HHV‐6A, potentially, by expanding the CTL and NK cell pool [206, 209]. Of particular interest is the complex interplay between CMV and EBV. Individuals with a CMV‐negative/EBV‐positive seroprofile face a significantly higher risk of MS development, suggesting that CMV may modulate the pathogenic potential of EBV [210, 211]. A presymptomatic cohort study demonstrated that CMV seropositivity correlated with a decreased risk of MS. Thus the absence of CMV infection may leave the immune system more vulnerable to the EBV‐associated autoimmune neuroinflammatory triggers [205].

In case of HIV, decreased risk of MS may be associated with immune suppression reducing autoimmunity and antiretroviral therapy inhibiting HERV expression.

### 5.3 EBV and HERVs

Viral interactions between HERVs and human herpesviruses elicit synergistic immune responses linked to autoimmune diseases, supporting the hypothesis that interactions between exogenous and endogenous viruses may facilitate the development of MS. Indeed, HERV‐W may have a pathogenic role in initiating and promoting MS [128]. In vitro studies using cells from MS patients have shown that antigens of EBV, HSV‐1, HHV‐6, and VZV can promote endogenous retroviral reverse transcriptase activity [127, 141]. In addition, clinical serological data revealed a correlation between HERV‐W/env antibody titres and EBV viral markers in MS patients [212].

The interaction between HERVs and EBV is a bidirectional process, where EBV infection or reactivation induces expression of HERV‐K18 and HERV‐W genes [213], thereby activating these endogenous retroviruses and inducing a cascade of inflammatory responses mediated by monocytes, T and B lymphocytes and NKs. Their associated cytokines IL‐1β, IL‐6 and TNF‐α are released as a secondary response leading to oligodendrocyte damage and demyelination [118, 214, 215]. On the other hand, HERV‐W‐env facilitates the ability of EBV to activate lytic replication [216]. Moreover, HERV‐W env capacity to elevate human iNOS expression, thereby contributing to BBB disruption, may facilitate the entry of EBV and other neurotropic viruses into the CNS, potentially amplifying the cascade of pathological immune responses associated with MS [217]. Indeed, a strong correlation between pHERV‐W‐env‐induced cytokine production and EBV and CMV titres was observed in MS patients [212].

HHV‐6A also activates HERVs [218]. HHV‐6 A directly *trans*‐activated HERV‐K18 through HHV‐6A produced IFN‐α [219]. HHV‐6 A induced the expression of MSRV‐Env through CD46 signalling while MSRV‐Env further triggered TLR4‐dependent pro‐inflammatory stimulation of immune cells and impairs human OPC maturation which drive MS pathogenesis [220, 221].

LTR‐directed transcription of the HERV‐W can be induced by the HSV‐1 infection in neuronal and brain endothelial cells in culture partly mediated by the action of the HSV‐1 immediate early protein 1 (IE1) [222]. HSV‐1‐induced HERV‐W gag and env expression in neuronal and brain endothelial cells may enhance their oligodendrotoxic and immunopathogenic effects in MS [223]. Additionally, HSV‐1 can also induce EBV lytic reactivation by initiating a signalling cascade wherein the viral kinase US3 activates host protein kinase A (PKA), which in turn phosphorylates CREB, enabling its binding to the promoter of the lytic transactivator gene *BZLF1* to initiate its transcription [140].

### 5.4 EBV and SARS‐CoV2

Viral co‐infections and reactivations subsequent to SARS‐CoV2 infection, result in pathogenic virus‐host interactions that may promote the onset or recurrence of autoimmune diseases [224]. More specifically, SARS‐CoV‐2‐induced immune suppression and hyper‐inflammation is frequently linked to EBV reactivation, which may occur shortly after or concurrently with SARS‐CoV‐2 infection, as well as following initially asymptomatic infections [225, 226]. Moreover, meta‐analysis data demonstrated that patients with severe COVID‐19 had a six‐fold increased likelihood of active EBV infection compared to non‐COVID‐19 controls [227] with a positive correlation with the severity of SARS‐CoV‐2 infection [228]. Recently, the incidence of HSV‐1, HSV‐2, CMV, and EBV reactivation was evaluated in critically ill COVID‐19 patients with EBV being the most frequently and earliest reactivated herpesvirus. Multiple viral reactivations occurred in 63% of patients [229]. However, this phenomenon may largely reflect lymphocytopenia, particularly in cases of severe diseases, creating a more permissive environment for viral reactivation. Furthermore, high‐dose corticosteroid therapy used to treat severe COVID‐19 may further exacerbate this immunosuppressed state. In addition, the critical condition of these patients, together with underlying conditions such as cancer or immunodeficiencies and their associated therapies, may further contribute to immune dysfunction. Therefore, viral reactivation in this setting is more likely to result from COVID‐19‐induced immune impairment rather than direct virus–virus interactions. In contrast, recent findings suggest that viral reactivation in COVID‐19 patients may also involve direct crosstalk between SARS‐CoV‐2 and latent herpesviruses beyond generalised immune dysfunction. The link between acute viral infections and their evident epidemiological association with MS may be explained by the possibility that herpesviruses take advantage of other acute illnesses to exit latency. EBV reactivation promotes the expression of the primary surface receptor angiotensin converting enzyme 2 (ACE2) that is involved in the SARS‐CoV‐2 entry into the epithelial cells since *ACE2* promoter contains response elements for EBV early lytic gene *BZLF1* further favouring EBV [230]. In addition, the hypoxia‐inducible factor 1α (HIF‐1α) that plays a key role in the virus infection and pro‐inflammatory responses promoting SARS‐CoV‐2 pathogenesis can directly bind *BZLF1* thereby initiating the EBV lytic cascade [231, 232].

Collectively, these findings indicate that SARS‐CoV‐2 mediated viral reactivation may result from general immune dysfunction, while also potentially involving mechanistic interactions between SARS‐CoV‐2 and latent EBV infection, with important implications for post‐viral autoimmunity and MS.

### 5.5 EBV and HIV‐1

Not all viral interactions have a negative effect on MS. Indeed, HIV‐1 targets CD4+ T lymphocytes, which are critically involved in the immunopathogenesis of MS [233]. HIV has a protective role in the development of MS, as there is a lower incidence of MS among individuals living with HIV than statistically expected [184, 186]. This negative association may be partly due to the ability of HIV to suppress the immune system, thereby preventing potential autoimmune responses [184]. However, the rate of MS was also significantly lower than expected after the first exposure to antiretroviral therapy (ART) [186]; this is particularly intriguing given that the treatment suppresses HIV replication and restores CD4+ cell counts, boosting immunity and increasing the capacity of EBV to trigger MS. This suggests additional complex mechanisms by which HIV may reduce the risk of MS. One proposed mechanism was the ability of ART to reduce the expression of HERVs, as HIV‐infected patients had lower expression of MSRV/HERV env than MS patients [234]. Protein interactions between HIV‐1 Tat and EBV have been reported, but they need to be investigated in MS context to uncover some molecular mechanisms involved in the protective effect, possibly leading to new therapy options [235].

Further studies are required to elucidate the specific molecular mechanisms of viral interaction, which is essential for validating their proposed involvement in the aetiology of neurological complications or MS‐related autoimmune processes. The molecular interactions between viruses identified to date are just a small part of this complex pathogenic network. A further characterisation of how these interactions contribute to the onset and advancement of MS is necessary for development of the next generation preventative and therapeutic strategies.

### 5.6 EBV and HTLV‐1

Despite the lack of a direct association between Human T‐lymphotropic virus type 1 (HTLV‐1) infection and MS, this virus serves as an important model for elucidating mechanisms of viral entry into the CNS, particularly via cellular trafficking across the BBB. (HTLV‐1) causes HTLV‐1‐associated myelopathy/tropical spastic paraparesis (HAM/TSP), a chronic inflammatory and progressive CNS disorder affecting ∼2% −3% of HTLV‐1‐infected individuals [236]. Both MS and HAM/TSP have similarities in CNS infiltration strategy: HLA‐mediated impairment of CTL response leads to failed control of the viral replication, subsequently allowing the infected cells to bypass the BBB and trigger neuroinflammation [237]. In both scenarios, the BBB crossing is not a viral event, but a cellular one. Specifically, HTLV‐1 hijacks CD4+ T‐cells, utilising the Tax protein to upregulate activated leucocyte cell adhesion molecule (ALCAM) that facilitates a ‘Trojan Horse’ entry into the CNS [238]. This mirrors the hypothesised trafficking in MS, where peripherally activated EBV‐primed lymphocytes exploit similar adhesive pathways, for example VLA‐4 upregulation, to enter the CNS. Furthermore, the resulting neurodegeneration in HAM/TSP is widely attributed to bystander damage rather than direct viral cytopathy or molecular mimicry, partially parallelling current views on EBV's role in MS‐related demyelination [239]. One important difference is that HTLV‐1 is detectable in CSF of HAM/TSP patients [240, 241], while the presence of EBV in MS demyelinating plaques is inconsistent, raising questions about its direct involvement [242, 243]. This suggests that EBV may play a key role in triggering autoimmunity and facilitating immune cell infiltration into the CNS, limiting the demyelination process solely to bystander damage.

## 6 Conclusions and Future Directions

The role of viral interactions in the etiopathogenesis MS is increasingly recognized. While EBV remains the central viral determinant, the cumulative findings demonstrate that MS cannot be fully explained by EBV infection alone. Rather, the disease appears to emerge from a multifactorial network of viral influences, involving both exogenous and endogenous agents that act in concert to sustain neuroinflammation and autoimmunity. Co‐infections and sequential reactivations among neurotropic viruses such as EBV, HHV‐6A, CMV, HSV‐1, VZV and HERVs can modulate one another's replication, immune activation patterns, and neuropathological consequences.

Several specific mechanistic links emerge from this analysis. HHV‐6 A has been shown to transactivate EBV lytic genes (notably BZLF1 lytic gene) and induce EBV latent proteins (LMP1, EBNA2), thereby enhancing B‐cell activation and autoantigen presentation. CMV seropositivity may exert a modulatory, potentially protective effect through competitive immune surveillance and NK‐cell reprogramming, suggesting that certain viral exposures can counterbalance pathogenic interactions. Furthermore, EBV and HERVs participate in a bidirectional relationship in which EBV reactivation upregulates HERV‐W and HERV‐K expression, while HERV‐W Env protein in turn facilitates EBV lytic activation and contributes to BBB disruption via TLR4‐and iNOS‐mediated pathways. The interplay between SARS‐CoV‐2 and EBV through ACE2 and HIF‐1α signalling further illustrates how acute viral infections may reactivate latent herpesviruses, amplifying autoimmune cascades.

From a translational perspective, these findings advocate for a paradigm shift from single‐agent causality models towards a systems‐virology framework, integrating the temporal, molecular, and immunological dimensions of multi‐viral interactions. Future research should aim to define the sequence and hierarchy of viral activations preceding MS onset using longitudinal cohort data and high‐sensitivity serological assays; map the inter‐viral regulatory networks at single‐cell resolution through transcriptomic and epigenomic profiling of immune and glial compartments; characterise virus‐induced perturbations in B‐cell repertoires and autoantibody landscapes; develop combinatorial antiviral and immunomodulatory strategies that target shared molecular pathways across herpesviruses and endogenous retroelements; evaluate EBV‐ and HHV‐6A‐specific vaccines and therapeutic monoclonal antibodies within this broader virological context.

In conclusion, MS may be better understood as the outcome of a dynamic virome–host interplay rather as a single infection‐driven disease. While current therapeutic approaches for established MS primarily target compartmentalised neurodegenerative process underlying progression independent of relapse activity (PIRA), translating our understanding of early pathogenic viral interactions into clinical practice remains a major challenge. Addressing this knowledge gap may provide new opportunities for identifying early predictive biomarkers and developing targeted strategies for the primary prevention of MS onset.

## Author Contributions

Y.V., E.D. and N.P.: conceptualisation. S.P., D.M., Y.V. and E.D.: writing the original draft of the paper with input from all co‐authors. S.P. and D.M.: visualisation. S.P., D.M., J.K., I.T., E.L., M.H., S.S., J.P., N.S., Y.V., E.D. and N.P.: writing – review and editing. Y.V. and N.P.: funding acquisition. All authors read and approved the final manuscript.

## Data Availability

Data sharing not applicable to this article as no datasets were generated or analysed during the current study.
